# Molecular evidence for sediment nitrogen fixation in a temperate New England estuary

**DOI:** 10.7717/peerj.1615

**Published:** 2016-01-25

**Authors:** Silvia E. Newell, Kaitlyn R. Pritchard, Sarah Q. Foster, Robinson W. Fulweiler

**Affiliations:** 1 Department of Earth and Environment, Boston University, Boston, MA, USA; 2 Department of Earth and Environmental Sciences, Wright State University, Dayton, OH, USA; 3 Department of Marine and Environmental Sciences, Northeastern University, Boston, MA, USA; 4 Department of Biology, Boston University, Boston, MA, USA

**Keywords:** *nifH*, Denitrification, Sediments, Sulfate-reducing bacteria, *nifH* diversity, Heterotrophic nitrogen fixation

## Abstract

Primary production in coastal waters is generally nitrogen (N) limited with denitrification outpacing nitrogen fixation (N_2_-fixation). However, recent work suggests that we have potentially underestimated the importance of heterotrophic sediment N_2_-fixation in marine ecosystems. We used clone libraries to examine transcript diversity of *nifH* (a gene associated with N_2_-fixation) in sediments at three sites in a temperate New England estuary (Waquoit Bay, Massachusetts, USA) and compared our results to net sediment N_2_ fluxes previously measured at these sites. We observed *nifH* expression at all sites, including a site heavily impacted by anthropogenic N. At this N impacted site, we also observed mean net sediment N_2_-fixation, linking the geochemical rate measurement with *nifH* expression. This same site also had the lowest diversity (non-parametric Shannon = 2.75). At the two other sites, we also detected *nifH* transcripts, however, the mean N_2_ flux indicated net denitrification. These results suggest that N_2_-fixation and denitrification co-occur in these sediments. Of the unique sequences in this study, 67% were most closely related to uncultured bacteria from various marine environments, 17% to Cluster III, 15% to Cluster I, and only 1% to Cluster II. These data add to the growing body of literature that sediment heterotrophic N_2_-fixation, even under high inorganic nitrogen concentrations, may be an important yet overlooked source of N in coastal systems.

## Introduction

Nitrogen (N) limitation in marine waters is controlled, in large part, by the balance between denitrification and nitrogen fixation (N_2_-fixation). While denitrification removes biologically reactive N from the environment, N_2_-fixation converts dinitrogen gas (N_2_) into biologically available ammonium. Although N_2_ is the most abundant element in the atmosphere, the majority of organisms on earth cannot use N in this form. Thus, N-fixing organisms, known as diazotrophs, are unique in that they can grow without external sources of reactive N by drawing upon the high concentrations of N_2_ dissolved in seawater (∼400–574 µmol L^−1^ N_2_: Sharp, 1983; Zehr & Paerl, 2008). Through the fixation of di-nitrogen gas, diazotrophs have the ability to increase reactive N availability and, subsequently primary and secondary production (Montoya, Carpenter & Capone, 2002; Ryther & Dunstan, 1971).

The genetic potential for N_2_-fixation is widespread among the Domains of Bacteria and Archaea (Zehr et al., 2003). In fact, geochemists argue that N cannot be the ultimate limiting nutrient, as diazotrophic microorganisms possess the ability to reduce N deficiencies given sufficient time (Redfield, 1958; Tyrrell, 1999). Despite this genetic potential, filamentous cyanobacteria (e.g., *Trichodesmium*) and diatom symbiotic cyanobacteria (e.g., *Richelia*) were traditionally thought to be the only marine diazotrophs that produce a significant source of new N to marine environments (Capone et al., 1997; Karl et al., 1997; Zehr et al., 2001). Additionally, N_2_-fixation was thought to be of only minor importance compared to N inputs from upwelling and advection of nitrate rich water in the open ocean (Zehr & Paerl, 2008) and river N loading in coastal systems (Howarth et al., 1988). Furthermore, heterotrophic sediment N_2_-fixation was generally considered to be of minimal significance to subtidal benthic N cycling, contributing as little as 0.1% of total new N to the environment (Howarth et al., 1988).

This view however, has changed dramatically over the last decade, with observations of significant N_2_-fixation by both unicellular autotrophic and heterotrophic diazotrophs in a range of marine environments. For example, unicellular cyanobacteria are now thought to contribute substantially to N_2_-fixation in temperate (Rees, Gilbert & Kelly-Gerreyn, 2009; Bentzon-Tilia et al., 2015b), subtropical (Zehr et al., 2001), and tropical (Goebel et al., 2007) water columns. Significant unicellular heterotrophic N_2_-fixation has also been reported in water columns from the Mediterranean Sea (Rahav et al., 2013), the Baltic Sea (Bostrom et al., 2007), and the hypoxic Southern California Bight (Hamersley et al., 2011). Additionally, N_2_-fixation has been found at hydrothermal vents (Mehta & Baross, 2006), in cold seep sediments (Dekas, Poretsky & Orphan, 2009), and associated with the decomposition of a large marine macrophyte (Hamersley et al., 2015). Recently, substantial fluxes of sediment N_2_-fixation has been observed through direct N_2_ uptake (Dias et al., 2012; Grosskopf et al., 2012; Rao & Charette, 2012) and *nifH* gene expression (Fulweiler et al., 2013; Andersson et al., 2014). In some cases, the reported sediment N_2_-fixation comprises a significant portion of the system N budget (Fulweiler & Heiss, 2014).

In order to expand our understanding of the potential role of N_2_-fixation as a nitrogen source in marine environments, we examined the expression of *nifH*, a gene encoding for nitrogenase (the enzyme associated with N_2_-fixation), in the sediments of a temperate New England estuary (Waquoit Bay, MA). The aim of this research was to investigate the spatial distribution of *nifH* transcript diversity at three sites with different environmental characteristics and to link these results to concurrently collected biogeochemical data. We had three specific questions: (1) Which organisms appear to be responsible for the observed *nifH* expression? (2) How did the active community composition and diversity change at these three estuarine sites? (3) Does *nifH* expression occur simultaneously with net sediment denitrification as has been reported for sediments in a nearby estuary (Fulweiler et al., 2013)? If so, this would indicate that these two opposite processes overlap in space and time highlighting the dynamic nature of the marine nitrogen cycle and how much more we need to learn about what controls its major processes. In turn, these findings will help us better inform estuarine N budgets, as we hypothesize that this process may provide a significant and yet overlooked source of N.

## Materials and Methods

### Description of study area

**Figure 1:**
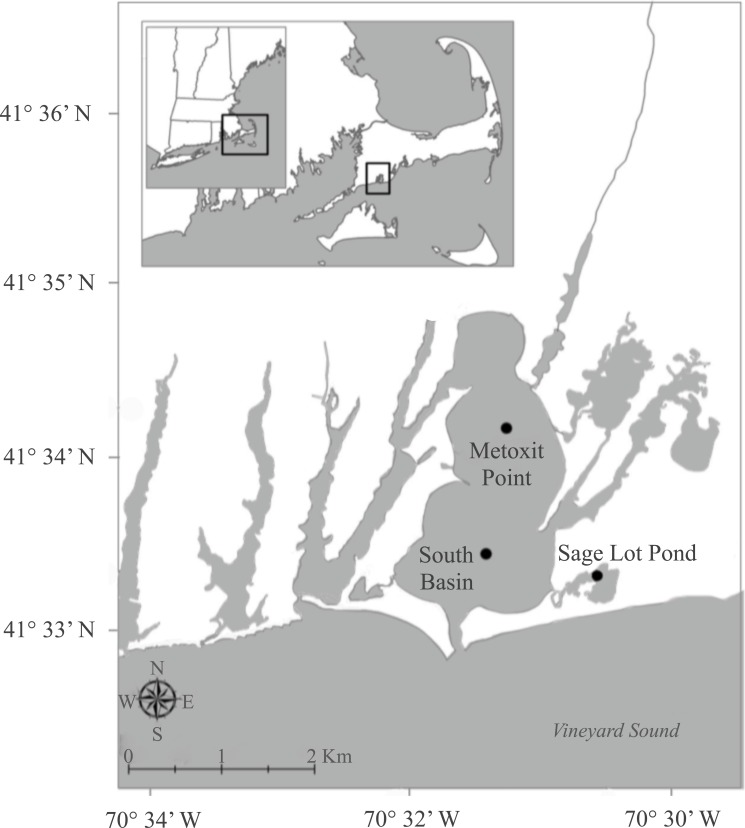
Map of sampling stations. The sampling sites where sediments were collected for *nifH* expression as part of a larger project (Foster & Fulweiler, 2014) focused on sediment nitrogen cycling in Waquoit Bay, Massachusetts.

Waquoit Bay is a shallow (mean depth 1.8 m) estuary on the southwestern shore of Cape Cod, Massachusetts (Fig. 1). Although the estuary is mostly landlocked, it opens to Vineyard Sound through a narrow (100 m wide) channel along its southern edge. Within Waquoit Bay we focused on three sites with varying *in situ* nutrient conditions and sediment net N_2_ fluxes (Table 1). Two of these sites, Metoxit Point (MP) and South Basin (SB), are located within the main estuary of Waquoit Bay and the third, Sage Lot Pond (SLP), is located just to the east (Fig. 1). MP is located directly downstream of the Quashnet River, which has a mean annual load of 50 × 10^3^ kg N km^−2^ y^−1^ (Bowen & Valiela, 2001; Foster & Fulweiler, 2014; Valiela et al., 1997). It is characterized as having extensive macroalgae blooms and extreme fluctuations in diel summer oxygen conditions. SB is located close to the mouth of the estuary where tidal flushing with Vineyard Sound water occurs and it is therefore presumably less impacted by anthropogenic N loading. Finally, SLP is our least N impacted site as it is located in an undeveloped, forested watershed and has a low annual N loading rate (2.1 × 10^3^ kg N km^−2^ y^−1^; Valiela et al., 1997; Valiela et al., 2000).

**Table 1:** Physical and biogeochemical characteristics for the Waquoit Bay sediment cores used for the molecular analyses in this study. Inorganic nutrient concentrations and salinity are from the water overlying each sediment core before the start of the incubations. The net N_2_–N fluxes across the sediment water interface are calculated for each core using a 5-point linear regression and rates were prorated for the volume and area of the cores. All data from Foster (2012) and Foster & Fulweiler (2014).

Site	Core-ID	Incubation date	Incubation temperature, °C	Salinity	${\mathrm{NH}}_{4}^{+}\mathrm{\mu }\mathrm{mol}\hspace{1em}{\mathrm{L}}^{-1}$	${\mathrm{NO}}_{2}^{-}+{\mathrm{NO}}_{3}^{-}\mathrm{\mu }\mathrm{mol}\hspace{1em}{\mathrm{L}}^{-1}$	${\mathrm{PO}}_{4}^{3-}\mathrm{\mu }\mathrm{mol}\hspace{1em}{\mathrm{L}}^{-1}$	Net N_2_–N flux μmol m^−2^ h^−1^
Metoxit Point	16	24-Aug-2011	25	30	11.35	0.11	0.58	−50
	7	11-Oct-2011	20	31	34.10	0.76	1.94	0
	18	11-Oct-2011	20	31	18.49	0.13	0.92	−51
	8	11-Oct-2011	20	31	98.91	0.30	5.36	nd
South Basin	9	7-Jul-2011	26	32	7.37	0.00	0.67	0
	1	24-Aug-2011	25	32	1.00	0.12	0.44	−51
	5	24-Aug-2011	25	32	1.87	0.15	0.41	28
	1	11-Oct-2011	20	31	nd	0.12	0.57	23
	6	11-Oct-2011	20	31	nd	0.12	0.54	75
	4	11-Oct-2011	20	31	nd	0.11	0.60	0
	5	11-Oct-2011	20	31	nd	0.12	0.49	15
Sage Lot Pond	8	29-Jul-2011	25	30	9.66	0.28	0.54	24
	8	24-Aug-2011	25	30	5.19	0.27	0.56	0
	19	24-Aug-2011	25	29	9.85	nd	0.76	191

**Notes.**

*nd* non-detect

### Sediment core collection and biogeochemical flux measurements

The research reported here was part of a larger study focused on sediment N cycling in Waquoit Bay (Foster, 2012; Foster & Fulweiler, 2014). Here we briefly describe the methods used in that study for core collection and biogeochemical flux measurements as they directly apply to this current research. Intact sediment cores (30 cm long, 10 cm diameter) were collected using a pole corer from each of three study sites (MP, SB, and SLP, Fig. 1): SLP in July and August 2011, and MP and SB in July, August and October 2011 (Table S1). At each station cores were collected within a meter of one another. These cores were kept cool and in the dark, transported to an environmental chamber at Boston University and held at ambient field temperatures (Foster, 2012; Foster & Fulweiler, 2014). The cores were left uncapped with air gently bubbling through the overlying water column overnight (10–12 h). The overlying water in each core was then carefully replaced with filtered (0.2 µm pore size) site water and sealed with a gas-tight lid (no air headspace) for dissolved inorganic nitrogen (DIN: ${\mathrm{NH}}_{4}^{+}$, ${\mathrm{NO}}_{2}^{-}+{\mathrm{NO}}_{3}^{-}$) and gas (N_2_ and O_2_) flux incubations (Foster & Fulweiler, 2014). Over the course of the incubations five water samples were taken from each core. For DIN samples the water was immediately filtered into 30 ml acid washed and deionized water leached polyetheylene bottles using an acid washed polypropylene syringe and glass fiber filters (Whatman GF/F, 0.70 µm pore size). These samples were frozen until analysis on a Seal Auto Analyzer 3, using standard colorimetric techniques (Grasshoff, Kremling & Ehrhardt, 2009). For the dissolved gas analysis water samples were analyzed for N_2_ and Ar using the N_2_∕Ar technique (Kana et al., 1994) and a membrane inlet mass spectrometer (MIMS, Bay Instruments). The N_2_∕Ar technique is a measure of net N_2_ production or consumption (gross denitrification minus gross N_2_-fixation). Over the course of the incubation the change in N_2_ concentration was determined from the change in the measured N_2_∕Ar multiplied by the known Ar concentration at air saturation (Colt, 1984). DIN and N_2_ flux across the sediment water interface for each core were calculated using a 5-point linear regression, and rates were prorated for the volume and area of the core.

### Sub-sampling for nucleic acid extractions

At the end of these incubations, we sub-cored the sediments at each site with modified sterilized 60 mL syringes. Samples were immediately frozen and stored at −80°C. For *nifH*expression, we extracted RNA from these cores at each station in 0.5 cm increments from 2 to 3 cm depth from our July and August sampling and from 1 to 3 cm from our October sampling (Table S1).

### RNA extraction and reverse transcription (RT)-PCR

RNA was extracted from ∼2.0 g of sediment using the MoBio RNA PowerSoil^®^ Total RNA Isolation Kit (Carlsbad, CA, USA). The RNA was purified using an Ambion TURBO DNA-free™ Kit (Austin, TX, USA) and was then used to synthesize cDNA with New England BioLabs Protoscript^®^ AMV First Strand cDNA Synthesis Kit (Ipswich, MA, USA). RNA and cDNA quality were confirmed visually via gel electrophoresis (large and small subunit, mRNA) and NanoDrop 260/280 (1.98-2.04 for RNA and 1.69-1.81 for cDNA) and 260/230 (2.19-2.31-1.98-2.19 for cDNA) ratios. RNA purity was also confirmed through addition as a template in *nifH* PCR. To prevent contamination, all work was performed in a PCR Hood (AirClean Systems, Raleigh, NC, USA) after the hood was decontaminated with a UV light (40 min) and RNase Away^®^ and DNA Away™ . All pipettes were also disassembled and cleaned with RNase Away^®^ and/or DNA Away™ before each use.

Multiple primer pairs were tested on our samples for reverse transcription PCR (RT-PCR) amplification, including IGK3-F and DVV-R (Ando et al., 2005), 19F and 388R (Ueda et al., 1995), 19F and nifH-univ-463r (Widmer et al., 1999), nested pairs nifH3 and 4 and *nifH* 1 and 2 (Zehr & McReynolds, 1989), and nifHPolF and nifHPolR primers (Poly et al., 2001). All primer pairs amplified the positive control, but only two sets amplified our samples as well. The nested (Zehr & McReynolds, 1989) primers produced optimum results, and were used for RT-PCR amplification followed by cloning. We performed a nested RT-PCR to amplify a 360-bp fragment of the *nifH* gene from environmental cDNA. 25 µL RT-PCR reaction mixtures containing 0.5 µL cDNA, 2.5 µL Mg^2+^-free 10X buffer, 1.5 µL MgCl_2_ (25 mM), 2 µL dNTP mixture (5 µM each), 1.5 µL each primer (10 µM), 14.5 µL nuclease-free water, and 1 µL Taq DNA polymerase (TaKaRa, Otsu, Japan) were amplified in a T100 Thermal Cycler (BioRad, Hercules, CA, USA). The first round RT-PCR, which used degenerate outer primers nifH3 (5′ ATRTTRTTNGCNGCRTA 3′) and nifH4 (5′ TTYTAYGGNAARGGNGG 3′) (Zehr & McReynolds, 1989), consisted of 25 cycling steps (1 min at 94°C, 1 min at 57°C, 1 min at 72°C) and a final 5 min extension step at 72°C (Zehr & McReynolds, 1989; Kirshtein, Paerl & Zehr, 1991). 0.5 µL of the first RT-PCR product was used as template in the second round RT-PCR, which used degenerate inner primers nifH1 (5′ TGYGAYCCNAARGCNGA 3′) and nifH2 (5′ ANDGCCATCATYTCNCC 3′) and consisted of 29 cycling steps with the same temperature profile. PCR-grade water (Ambion, Waltham, MA, USA) was used as a negative control and carried over from the first to second step of the RT-PCR, to ensure no contaminant was amplified.

### Cloning and nucleotide sequencing

We visualized the amplified *nifH* fragments using gel electrophoresis on 1% agarose gels stained with ethidium bromide. *nifH* bands were excised and cleaned using a QIAquick Gel Extraction Kit (Qiagen, Valencia, CA, USA). *nifH* amplicons from multiple depths were combined within some samples. Gel purification was performed according to the manufacturer’s specifications but included 3 additional ethanol rinses. Cleaned fragments were then immediately inserted into a pCR^®^ 2.1-TOPO^®^ vector using One Shot^®^ TOP10 Chemically Competent E. coli cells and a TOPO TA Cloning^®^ Kit (Invitrogen, Leek, The Netherlands). Insert-containing white colonies were randomly selected and sequenced at the GENEWIZ DNA sequencing facility (http://www.genewiz.com/) in Cambridge, MA using M13 forward (−20) and M13 reverse primers.

### Phylogenetic analysis

We assembled, edited, and aligned *nifH* nucleotide sequences in Geneious v.6.1.5 (Biomatters Ltd.) and checked for identity in the NCBI BLAST database (July 10, 2014). Waquoit Bay expressed *nifH* sequences and relevant published *nifH* sequences were then aligned (with a 65% similarity cost matrix) with MUSCLE (Edgar, 2004), a multiple sequence alignment tool, bootstrapped 1,000 times. A maximum likelihood protein tree was then constructed using Mega 6.0 (Tamura, 1992; Tamura et al., 2011; Hall, 2013), and bootstrap analysis with 1,000 replicates was used to estimate the accuracy of phylogenetic reconstruction (e.g., Mills et al., 2008; Hall, 2013). Original *nifH* cDNA sequences from this study have been deposited in GenBank under the accession numbers KF662225– KF662315and KM485700– KM485908.

### Statistical analysis

Operational taxonomic units (OTUs) were defined as *nifH* sequence groups in which sequences differed by ≤5% and ≤15% over the aligned 360-bp region (Geneious alignment) using the MOTHUR 10.6 program (Schloss et al., 2009; Table S1). We used MOTHUR 10.6 to calculate the non-parametric estimate of the Shannon diversity index, which is used when the number of species and the species abundances are unknown and there are a significant number of undetected species (Chao & Shen, 2003), and the Simpson’s evenness calculation, which is also less impacted by sample size, because it is weighted toward the most abundant OTUs (Hughes & Bohannan, 2004). MOTHUR was also used to determine the Morisita–Horn index, which provides a (dis)similarity measurement based on presence–absence data only (Magurran, 1988) and is not influenced by sample size or richness (Wolda, 1981). Rarefaction analysis was also performed in MOTHUR. All tests were performed for OTU groups at both the 5% and 15% cutoff levels. All figures and tables in the main text include both the 5% and the 15% OTU cutoff values. *nifH* transcript abundance data were log-transformed for normalization and differences among sites were determined by one-way ANOVA and paired Student’s *t*-test.

**Figure 2:**
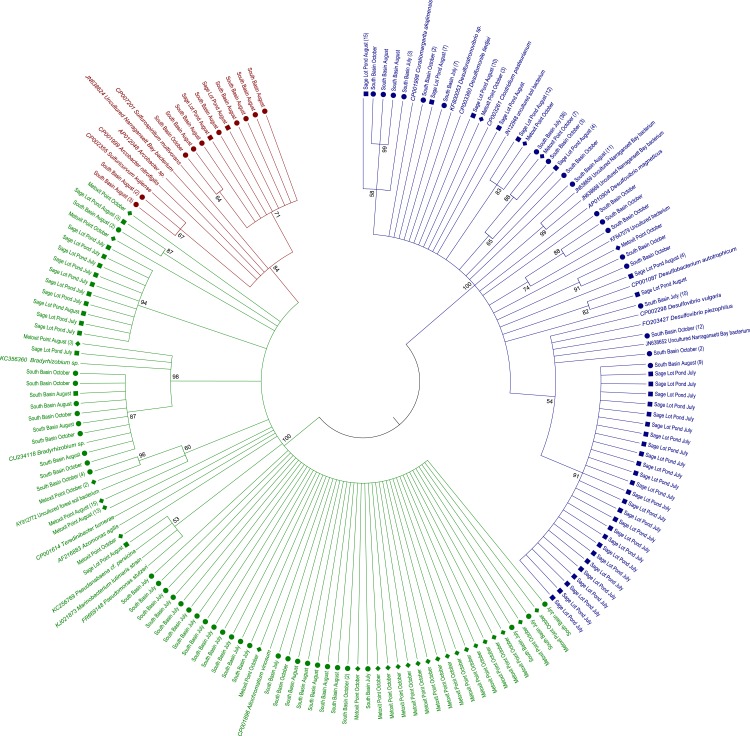
Maximum likelihood amino acid tree of expressed *nifH* sequences in sediment samples from this study as well as *nifH* sequences from cultured and other environmental sample representatives. Sequences are highlighted by shape for location: North Basin (diamonds), South Basin (circles), and Sage Lot Pond (squares). Where we collapsed branches the number of sequences collapsed is given in parentheses. Sequences in green are from *nifH* Cluster 1, red Cluster II, and blue Cluster III. All accession numbers can be found in Table S1. The evolutionary history was inferred by using the Maximum Likelihood method based on the JTT matrix-based model (Jones, Taylor & Thornton, 1992). The bootstrap consensus tree inferred from 1,000 replicates (Felsenstein, 1985) is taken to represent the evolutionary history of the taxa analyzed (Felsenstein, 1985). Branches corresponding to partitions reproduced in less than 50% bootstrap replicates are collapsed. The percentage of replicate trees in which the associated taxa clustered together in the bootstrap test (1,000 replicates) are shown next to the branches (Felsenstein, 1985). Initial tree(s) for the heuristic search were obtained automatically by applying Neighbor-Join and BioNJ algorithms to a matrix of pairwise distances estimated using a JTT model, and then selecting the topology with superior log likelihood value. The analysis involved 334 amino acid sequences. All positions containing gaps and missing data were eliminated. There were a total of 93 positions in the final dataset. Evolutionary analyses were conducted in MEGA6 (Tamura et al., 2013).

**Figure 3:**
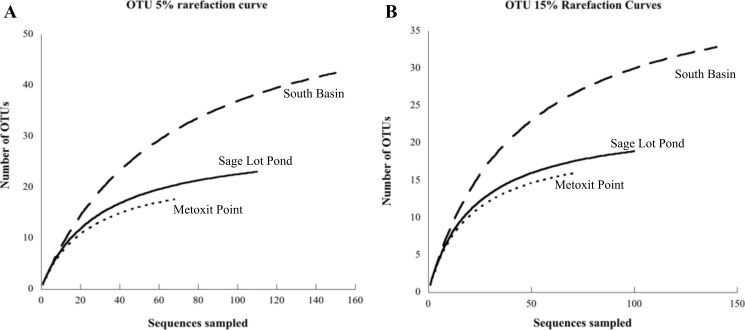
Rarefaction curves for each site. Each curve displays the number of unique OTUs versus the number of *nifH* clones sequenced from sediment samples at each site (Metoxit Point dotted line, South Basin dashed line, and Sage Lot Pond solid line). OTUs are defined according to a 95% cutoff (A) or an 85% cutoff (B). All rarefaction values were calculated in MOTHUR (Schloss et al., 2009).

**Table 2:** Diversity and evenness indices for each site.

	Metoxit Point	South Basin	Sage Lot Pond
*5% cutoff*			
Non-parametric Shannon diversity	2.75	3.65	2.94
Simpson evenness	0.76	0.63	0.62
Morisita–Horn index	1	1	0.99
*15% cutoff*			
Non-parametric Shannon diversity	2.6	3.32	2.76
Simpson evenness	0.7	0.66	0.74
Morisita–Horn index	0.75	0.8	0.7

**Figure 4:**
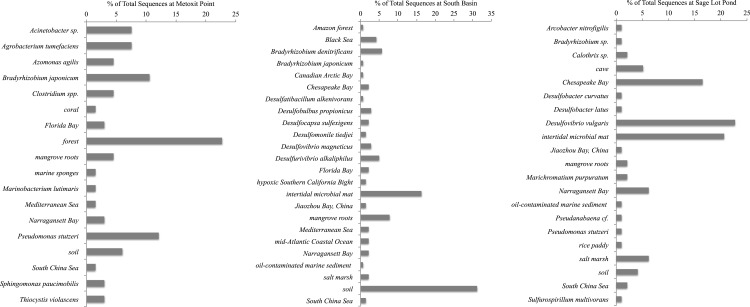
Most closely related organisms from the NCBI database to *nifH* sequences from the three study sites (Metoxit Point, South Basin, and Sage Lot Pond) in Waquoit Bay.

## Results

Of the 18 cores analyzed, *nifH* expression was detected and ultimately sequenced from 14 cores (3, 8, 3 from MP, SB, and SLP, respectively), revealing diverse *nifH* expression in Waquoit Bay sediments. In total, we successfully sequenced 307 cDNA clones. Of these, 289 were unique sequences, which phylogenetically grouped into three of the major clusters (Cluster I, II, and III) previously used to characterize *nifH* phylogeny (Chien & Zinder, 1994; Fig. 2). The 5% sequence variation was selected for the operational taxonomic unit (OTU) cut-off to analyze phylotype richness and diversity of *nifH* bacteria found at each site. 85 OTUs were identified at the 5% cutoff, with most expressed sequences grouping together by date and site location (Table S1). South Basin showed the greatest diversity (non-parametric Shannon diversity was 3.65) of the three sites, and it also had the greatest number of unique sequences (133 vs. 55 at MP and 101 at SLP) (Table 2). The active *nifH* community at MP was the least diverse (2.75). Rarefaction analysis showed the greatest richness to be at SB, with MP and SLP less rich (Fig. 3). A significant difference was detected between SB (the most diverse and rich) and MP (the least diverse and rich site with the fewest unique sequences) at both the 5% and 15% cutoffs (ANOVA, *p* = 0.01 and *p* = 0.02 respectively). The Morisita–Horn index, indicating dissimilarity between communities, shows very different communities at the 5% cutoff between all three sites, as there was only a single shared OTU (Table 2). However, at the 15% cutoff, greatest dissimilarity is between MP and SLP (Morisita–Horn value of 0.80), while the greatest similarity (lowest Morisita–Horn value) is between SB and SLP (0.70) (Table 2).

Expressed *nifH* sequences at each site were distributed throughout our phylogenetic tree (Fig. 2). MP *nifH* sequences were primarily contained in Cluster I. The largest OTU in MP (7) contained sequences closely related (87–94% similar) to other uncultured *nifH* sequences from forest soil (Rosch & Bothe, 2009). The second and third largest OTUs (15 and 17) contained sequences most closely related (87–93% similar) to *Acinetobacter* sp. Z21 (EU693341), *Agrobacterium tumefaciens* (FJ822995) *nifH*, and *Bradyrhizobium japonicum* (GQ289582) (Fig. 4). We also observed the presence of a close relative (88–93% similar) of *Thiocystis violascens*, a purple sulfur bacteria that can grow with sulfide or sulfur as an electron donor under anoxic conditions (Lucas et al., 2012). MP *nifH* sequences were also >87% similar to sequences from uncultured bacteria found in a wide range of environments, including the Mediterranean Sea (Yogev et al., 2011), mangrove roots (Flores-Mireles, Winans & Holguin, 2007), and soil (Berthrong et al., 2014).

SB *nifH* sequences were most closely related to uncultured bacteria and Cluster II or Cluster III sequences. The largest SB OTU (3) encompassed sequences most closely related (82–95% similar) to sequences from uncultured bacteria from soil (Lopez-Lozano et al., 2012) and a Dutch intertidal microbial mat (Severin & Stal, 2010). The second largest OTU (4) contained sequences related (89–93% similar) to *Desulfovibrio* sp. and *Desulfobulbus* sp. *nifH* sequences (Fig. 4). One major OTU (9) did contain Cluster 1 sequences, most closely related (98–99% similar) to the *nifH* sequences from *Bradyrhizobium denitrificans*. Several of the small OTUs also contained *Desulfo*-related sequences.

Sequences from SLP were largely related to Cluster III sequences or uncultured bacteria. The two largest OTUs of all were comprised of SLP sequences most closely related (87–93% similar) to *nifH* sequences from uncultured bacteria from similar environments: Narragansett Bay (Fulweiler et al., 2013), Chesapeake Bay (GenBank Accession Number DQ098254), and a Dutch intertidal microbial mat (Severin & Stal, 2010). The third largest SLP OTU exclusively contained sequences related to *nifH*sequences (85–96% similar) from *Desulfovibrio vulgaris* (Fig. 4). Both SB and SLP also contained *nifH* transcripts similar to those of the sulfur-oxidizing *Sulfiricurvum kujiense* and sulfur-reducing *Sulfurospirillum multivorans* from Cluster II (92% similar).

**Figure 5:**
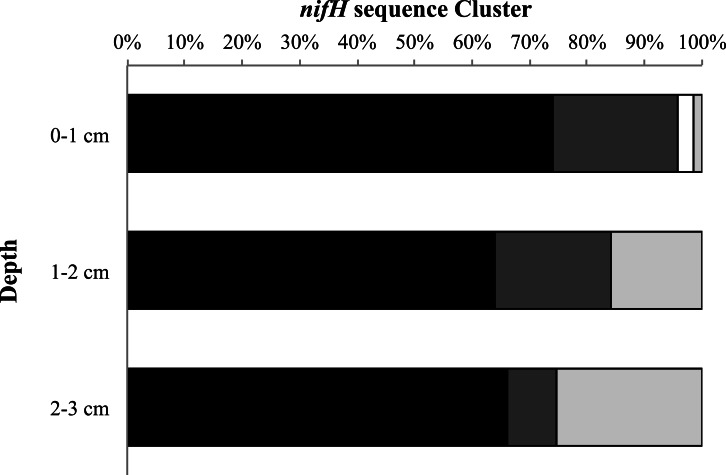
*nifH* cluster identity with depth. Cluster identity with depth for Metoxit Point, South Basin, and Sage Lot Pond for August 2011 (the month with the best sequence coverage at each depth). Dark gray represents Cluster I, light gray represents Cluster II, black represents Cluster III, and white represents unknown, uncultured matches. Cluster identifications were based on closest BLAST matches from the NCBI database.

Finally, we examined the relative percentage of uncultured as well as Cluster I, II, and III sequences for all sampling dates and stations as a function of depth (Fig. 5). We found the highest relative percentage of uncultured bacteria in the surface (0–1 cm) sediments and the highest percentage of Cluster III sequences deeper in the sediments (2–3 cm). For all sampling dates there is a trend of increasing relative abundance of Cluster III sequences accounting for more of the sequences with depth, from 1% at the surface to 25% at depth (Fig. 5).

## Discussion

N-fixing microorganisms from Clusters I, II, and III were found in Waquoit Bay sediments, illustrating the diversity of taxa containing *nifH* sequences, although the gene itself has been well-conserved. The majority of the sequences were most closely related to uncultured environmental sequences. It is worth noting that Cluster II nitrogen fixers are rarely reported in environmental samples (Zehr et al., 2003), yet we found they accounted for ∼3% of the surface sediment sequences (Fig. 5). Organisms falling into this cluster have alternative nitrogenases and maybe associated with methanogenic archaea (Zehr et al., 2003; Dang et al., 2009). Their presence is interesting and suggests these types of organisms can play a key role in environmental nitrogen cycling.

At all stations, we detected the presence of bacteria involved in sulfur cycling. For example, sequences similar to *Desulfovibrio* sp., a Cluster III sulfate reducing bacteria (SRB), were observed at SB and SLP. And, at MP, we observed *Thiocystis violascens*, which can grow photolithoautrophically using sulfide, sulfur, and occasionally thiosulfate (Lucas et al., 2012). Over six decades ago, Sisler & ZoBell (1951) measured N_2_-fixation via direct N_2_ uptake in *Desulfovibrio*. Since then, SRB have been found fixing N in a variety of environments, including in the above- and below-ground biomass of salt marshes (Lovell & Davis, 2012), seagrass beds (Welsh et al., 1996), microbial mats (Steppe & Paerl, 2002), sediments associated with ghost shrimp *Neotrypaea califoriensis* (Bertics et al., 2010), and temperate estuarine sediments (Fulweiler et al., 2013). *Desulfovibrio*-related phylotypes were also found associated macroalgae covered water columns (Zhang et al., 2015). Heterotrophic N_2_-fixation has been observed in the anoxic, ammonium rich waters of the Baltic (Farnelid et al., 2013). Our findings in this study are similar to this recent work. Specifically, at MP, the site with the highest *in situ* water column ammonium concentrations (Table 1) and significant macroalgae blooms, *nifH* expression was detected at all depths. Additionally, this site had an overall mean net N_2_ uptake −34 ± 16 µmol N_2_-Nm^−2^y^−1^ (*n* = 4), indicating net N_2_-fixation (Foster & Fulweiler, 2014). This was the only site to exhibit overall mean net N_2_-fixation. This site is closest to a recent study in Waquoit Bay that also observed N_2_-fixation in sandy, organic rich sediments in along the north shore of the main basin (Rao & Charette, 2012). The majority of uncultured matches were from soil clones (Rosch & Bothe, 2009), while many of the Cluster I cultured matches were from organisms with the genetic capability to both fix N and denitrify (e.g., *Pseudomonas stutzeri* and *Bradyrhizobium* sp.). The active *nifH* community at this site was the least diverse (non-parametric Shannon diversity = 2.75) and rich. We propose that the organic-rich environment is promoting a small number of dominant, actively N-fixing organisms. Although some studies have reported higher *nifH* gene diversity in highly N-impacted, organic rich sites (e.g., Chesapeake Bay: Moisander et al., 2007), a study of Danish estuaries with high nutrient loading showed greater *nifH* diversity at the DNA-level, but generally lower diversity in expressed *nifH* transcripts (Bentzon-Tilia et al., 2015b). Thus, nitrogen fixers have a diverse approach for dealing with environmental conditions (Bentzon-Tilia et al., 2015a).

These findings fit well with a recent turning of the tide in our understanding of estuarine nitrogen fixation. Historically, low rates of estuarine N_2_-fixation have been attributed to a variety of biogeochemical and physical mechanisms (Howarth, Marino & Cole, 1988a), as well as to the presence of inorganic nitrogen. Specifically, estuarine sediments are typically replete with biologically useable nitrogen such as ammonium, which is known to shut-off N_2_-fixation in cyanobacterial diazotrophs (Howarth, Marino & Cole, 1988a). However, a recent review highlighted that diazotroph sensitivity to inorganic nitrogen appears to vary with both the diazotroph type and the geographic location of the N_2_-fixation (Knapp, 2012). In fact, Knapp (2012) reports significant N_2_-fixation at ammonium concentrations as high as 200 µM, and it appears that benthic N-fixers may be much less sensitive to inorganic nitrogen exposure. Although Andersson et al. (2014) report an overall negative impact of dissolved inorganic nitrogen on nitrogenase activity, they did observe substantial rates of nitrogenase activity at ammonium concentrations up to 350 µmol L^−1^. In recent culture experiments of heterotrophic water column nitrogen fixers, Bentzon-Tilia et al., 2015a; Bentzon-Tilia et al., 2015b found that bacteria isolates had different responses to environmental characteristics. In particular, they report increased ammonium concentrations stimulate N_2_-fixation of one isolate (*Rhodopseudomonas palustris*, strain BAL398). They propose that this organism is using nitrogenase as an electron sink. Thus, other heterotrophic sediment bacteria may be employing N_2_-fixation for a similar reason.

Overall, our findings at the heavily N impacted site, MP, support the idea that at least some benthic, heterotrophic N fixers are less sensitive to dissolved inorganic nitrogen exposure. Specifically, we observed net N_2_-fixation at this site in addition to the highest *in situ* water column ammonium concentrations (98.91 µmol L^−1^) (Table 1). Other work has also reported similar findings. For example, research in the Chesapeake Bay found the highest *nifH* richness at the site most heavily impacted by N (Moisander et al., 2007). And high rates of directly measured N_2_-fixation have been reported for the nearby Providence River Estuary, another highly N impacted estuarine site (Fulweiler et al., 2007). We hypothesize that in the case of MP, sediment N_2_-fixation is occurring at a fast enough and high enough rate to be adding new nitrogen to the system. This is supported by recent experimental work in Waquoit Bay at this same site where ^30^N_2_ was fixed and later released as ^15^N-labeled ammonium (SE Newell et al., 2016, unpublished data). If these findings were confirmed in other systems, it would suggest that subtidal heterotrophic N_2_-fixation should be considered more often in coastal sediment N budgets. Additionally, it highlights the complex dynamics controlling sediment N_2_-fixation and, along with the Knapp (2012) review calls into question the idea that ammonium alone controls rates of sediment N_2_-fixation.

The other two, less N impacted sites (SB and SLP) also exhibited *nifH*expression, including Cluster III SRB. In contrast to MP, the mean sediment net N_2_ fluxes at SB and SLP in 2011 were positive, indicating net denitrification. SLP exhibited the highest net denitrification rate of 191 µmol N_2_–Nm^−2^h^−1^, followed by 75 µmol N_2_-Nm^−2^h^−1^ at SB. On one occasion (August 2011), a core at SB exhibited net N_2_-fixation (−51 µmol N_2_-Nm^−2^h^−1^). Thus, even though *nifH* was active and, as shown in August for SB N_2_-fixation was likely occurring, denitrification dominated. This signal could be driven by the spatial distribution of microbes in the sediment. For example, if denitrification occurred in the sediment surface, closer to the sediment-water interface (Froelich et al., 1979) and the SRB N_2_-fixation below, our gas fluxes might be capturing more of the surface net denitrification signal. In fact, co-occurring spatial and temporal *nifH* and *nirS* (a gene encoding for denitrification) expression along with directly measured rates of denitrification was recently reported in sediments of a nearby estuary (Narragansett Bay, RI, USA; Fulweiler et al., 2013). And Brown & Jenkins (2014) observed concurrent *nifH* and *nirS* expression along a high to low N loading gradient in Narragansett Bay. Together these findings suggest that these two opposing processes, N_2_-fixation and denitrification, may routinely co-occur in estuarine sediments. Fulweiler et al. (2013) proposed that while these processes may co-occur they may not be coupled but rather competitive, with N-fixers being able to outcompete denitrifiers for poor quality carbon sources. We also detected sequences similar to those from bacteria that have the capacity to both fix N and denitrify, such as *Pseudomonas stutzeri* and *Bradyrhizobium denitrificans*. These results are similar to a recent study of estuarine water column N_2_-fixation where *Bradyrhizobium*, *Pseudomonas*, and *Desulfovibrio* were present (Bentzon-Tilia et al., 2015b). Together, these studies suggest that we need to reframe the way we think about estuarine N_2_-fixation as the presence and activity of these organisms are found throughout these systems and likely play a key role in estuarine N budgets. Learning how diazotrophs and denitrifiers interact is a key next step toward understanding N limitation in coastal systems.

Of course a key question still remains: since N_2_-fixation is considered to be a metabolically costly process, requiring 16 ATP per N_2_ reduced, why fix nitrogen in an N-replete environment like an estuary? First, it is possible that nitrogenase may act as an electron sink for certain heterotrophic bacteria. In such a case they are not fixing N to obtain nitrogen but are using it as mechanism to cope with environmental conditions (McKinlay & Harwood, 2010; Bentzon-Tilia et al., 2015a). Additionally, we propose that in many ways sediments may provide an ideal environment for heterotrophic N_2_-fixation. One of the primary reasons why N_2_-fixation is so energetically expensive is the cost of keeping the highly sensitive nitrogenase enzyme away from oxygen. But most estuarine sediments are anoxic within the top few millimeters thus relieving this constraint and decreasing the overall energy requirements (Paerl & Prufert, 1987; Beman, Popp & Alford, 2012; Bonaglia et al., 2014). Additionally, the variety of marine environments that have shown linkages between N_2_-fixation and sulfur and sulfate reducers suggest that conditions that promote sulfur cycling might also promote N_2_-fixation (Sisler & ZoBell, 1951; Welsh et al., 1996; Fulweiler et al., 2013). Estuarine sediments are key environments for sulfur and sulfate reduction as they are anoxic, rich in organic matter, and have an abundant supply of sulfate ions from the surrounding seawater. Finally, trace metals critical to N_2_-fixation, such as iron and molybdenum, are abundant in estuarine sediments and thus likely do not limit N_2_-fixation as they may in estuarine water columns (Howarth, Marino & Cole, 1988a).

## Conclusions

We measured *nifH* expression in the sediments at every site sampled, even when net N_2_ fluxes indicated that denitrification was the dominant N_2_ pathway. These findings add to a growing body of evidence that suggest these opposing processes, N_2_-fixation and denitrification, may routinely co-occur in estuarine sediments. We observed the presence of sulfate reducing bacteria (SRB), as well as bacteria that have the capacity to both fix N and denitrify, such as *Pseudomonas stutzeri* and *Bradyrhizobium denitrificans*. The observations of *nifH* expression occurring with net denitrification, combined with presence of organisms that can both denitrify and fix nitrogen, suggest that the balance between those two processes may occur on the individual cellular level. The research presented here highlights a new perspective emerging from estuarine sediment N_2_-fixation studies in which the nitrogen, sulfur, and carbon cycles in coastal marine ecosystems are intimately connected. Additionally, it highlights that heterotrophic N_2_-fixation is an important and perhaps too often overlooked source of reactive N in coastal ecosystems. Future studies should also include measuring water column heterotrophic N_2_-fixation as recent studies have shown its importance and thus it too may play a critical role in estuarine N budgets.

## Supplemental Information

10.7717/peerj.1615/supp-1 Table S1 Sequences, accession number, and most closely related organism for all dat used in this study

## Additional Information and Declarations

### DNA Deposition

The following information was supplied regarding the deposition of DNA sequences: GenBank: accession numbers KF662225– KF662315and KM485700– KM485908.
