# Supplementary material for: Molecular evidence for sediment nitrogen fixation in a temperate New England estuary
Source: PeerJ. 2016 Jan 25;4:e1615. doi: 10.7717/peerj.1615 (PMC4788212; doi:10.7717/peerj.1615)
Supplement: Table S1 [file peerj-04-1615-s001.pdf]

Sample sequence	Site	.OTU	Accession Number	% Pairwise Identity	Organism	Author
MP11OctnifH-11-61_A07	MP	17	GQ289582	89	Bradyrhizobium japonicum	
MP11OctnifH-12-61_B07	MP	17	GQ289582	89.1	Bradyrhizobium japonicum	
MP11OctnifH-13-61_C07	MP	22	KJ021873	0.9	Marinobacterium lutimaris	
MP11OctnifH-14-61_D07	MP	25	FR669144	89.7	Pseudomonas stutzeri	
MP11OctnifH-15-61_E07	MP	17	GQ289583	89.6	Bradyrhizobium japonicum	
MP11OctnifH-16-61_F07	MP	22	FR669144	94.6	Pseudomonas stutzeri	
MP11OctnifH-17-61_G07	MP	25	FR669144	94.6	Pseudomonas stutzeri	
MP11OctnifH-18-61_H07	MP	22	FR669144	94.6	Pseudomonas stutzeri	
MP11OctnifH-20-61_B08	MP	25	FR669144	94.6	Pseudomonas stutzeri	
MP11OctnifH-21-61_C08	MP	25	HM601490	95	Florida Bay	Olson and Lesser, unpublished
MP11OctnifH-22-61_D08	MP	22	FR669144	94.6	Pseudomonas stutzeri	
MP11OctnifH-24-61_F08	MP	25	HM601490	90.1	Florida Bay	Olson and Lesser, unpublished
MP11OctnifH-25-61_G08	MP	22	FR669144	94.6	Pseudomonas stutzeri	
MP11OctnifH-26-61_H08	MP	22	FR669144	94.6	Pseudomonas stutzeri	
MP11OctnifH-27-61_E06	MP	17	GQ289582	89.6	Bradyrhizobium japonicum	
MP11OctnifH-28-61_F06	MP	17	GQ289582	78.1	Bradyrhizobium japonicum	
MP11OctnifH-29-61_G06	MP	17	GQ289582	89.6	Bradyrhizobium japonicum	
MP11OctnifH-30-61_H06	MP	17	GQ289582	89.2	Bradyrhizobium japonicum	
MP18-11Oct_0412A	MP	41	HQ606017	99.7	Mediterranean Sea	Yogev et al. 2011 et al.
MP18-11Oct_0412B	MP	41	KF182367	99.3	Sphingomonas paucimobilis	
MP18-11Oct_0412C	MP	41	KF182367	99.3	Sphingomonas paucimobilis	
MP18-11Oct_0614A	MP	84	DQ177000	83.7	Mangrove roots	Flores-Mirales et al. 2007
MP18-11Oct_0614B	MP	20	KF846744	94.4	soil	Berthrong et al 2014
MP18-11Oct_0614C	MP	20	KF846744	94.4	soil	Berthrong et al 2014
MP18-11Oct_0614D	MP	20	DQ177021	89.2	Mangrove roots	Flores-Mirales et al. 2007
MP18-11Oct_0614E	MP	20	DQ176983	89.2	Mangrove roots	Flores-Mirales et al. 2007
MP18-11Oct_0614F	MP	20	CP003154	88.6	Thiocystis violascens	
MP18-11Oct_0614G	MP	20	CP003154	93.3	Thiocystis violascens	
MP24AugnifH-38-432_A08	MP	23	AY912772	88	forest	Rösch & Bothe 2009
MP24AugnifH-39-432_B08	MP	7	AY912772	88	forest	Rösch & Bothe 2009
MP24AugnifH-40-432_C08	MP	7	AY912772	88	forest	Rösch & Bothe 2009
MP24AugnifH-41-432_D08	MP	7	AY912562	93.3	forest	Rösch & Bothe 2009
MP24AugnifH-42-432_E08	MP	15	EU693341	93.6	Acinetobacter sp. Z21	
MP24AugnifH-43-432_F08	MP	15	EU693341	93.3	Acinetobacter sp. Z21	
MP24AugnifH-44-432_G08	MP	15	FJ822995	93.5	Agrobacterium tumefaciens	
MP24AugnifH-45-432_H08	MP	15	FJ822995	93.5	Agrobacterium tumefaciens	
MP24AugnifH-46-432_A09	MP	15	FJ822995	87.5	Agrobacterium tumefaciens	
MP24AugnifH-47-432_B09	MP	21	AF216883	91.4	Azomonas agilis	
MP24AugnifH-48-432_C09	MP	7	AY912772	93.9	forest	Rösch & Bothe 2009
MP24AugnifH-49-432_D09	MP	15	EU693341	93.3	Acinetobacter sp. Z21	
MP24AugnifH-50-432_E09	MP	7	AY912562	87.5	forest	Rösch & Bothe 2009
MP24AugnifH-51-432_F09	MP	21	FJ822995	93.5	Agrobacterium tumefaciens	
MP24AugnifH-52-432_G09	MP	7	AY912772	88	forest	Rösch & Bothe 2009
MP24AugnifH-53-432_H09	MP	23	AY912772	88.2	forest	Rösch & Bothe 2009
MP24AugnifH-54-432_A10	MP	7	AY912772	87.8	forest	Rösch & Bothe 2009
MP24AugnifH-55-432_B10	MP	23	AY912566	93.3	forest	Rösch & Bothe 2009
MP24AugnifH-56-432_C10	MP	15	EU693341	93.8	Acinetobacter sp. Z21	
MP24AugnifH-57-432_D10	MP	7	AY912772	87.8	forest	Rösch & Bothe 2009
MP24AugnifH-58-432_E10	MP	21	EU693341	88	Acinetobacter sp. Z21	
MP24AugnifH-59-432_F10	MP	21	AF216883	91.4	Azomonas agilis	
MP24AugnifH-60-432_G10	MP	7	AY724202	93.3	forest	Rösch & Bothe 2009
MP24AugnifH-61-432_H10	MP	7	AY912562	87.2	forest	Rösch & Bothe 2009
MP24AugnifH-62-432_A11	MP	21	AF216883	89.6	Azomonas agilis	
MP24AugnifH-7-432_C10	MP	23	AY912772	88	forest	Rösch & Bothe 2009
MP24AugnifH-8-432_D10	MP	23	AY912772	87.2	forest	Rösch & Bothe 2009
MP24AugnifH-9-432_E10	MP	21	FJ822995	93.6	Agrobacterium tumefaciens	
MP7-11Oct_0625A	MP	84	KF861243	0.847	soil	Berthrong et al 2014
MP7-11Oct_0625B	MP	80	EU594035	84	marine sponges	Mohamed et al. 2008
MP7-11Oct_0625C	MP	83	HQ455977	88.1	South China Sea	Wu et al., unpublished
MP7-11Oct_0625D	MP	42	JN645336	94.8	Narragansett Bay	Fulweiler et al. 2013
MP7-11Oct_0625E	MP	81	KF847143	0.849	soil	Berthrong et al 2014
MP7-11Oct_0625F	MP	42	JN645336	92.4	Narragansett Bay	Fulweiler et al. 2013
MP7-11Oct_0703A	MP	37	AY603957	90.6	Clostsridium pasteurianum	
MP7-11Oct_0703B	MP	37	CP000721	82.4	Clostridium beijerinckii	
MP7-11Oct_0703C	MP	37	AY603957	81.2	Clostsridium pasteurianum	
MP7-11Oct_0703D	MP	82	KF657141	92.9	Coral	Santos, unpublished
SB1-11Oct_0625A	SB	4	GU193560	92.8	intertidal microbial mat	Severin & Stal 2010
SB1-11Oct_0625B	SB	4	GU193560	90	Desulfobulbus propionicus	
SB1-11Oct_0625C	SB	4	GU193539	90.3	Desulfobulbus propionicus	
SB1-11Oct_0625D	SB	4	JN645436	89.7	Desulfurivibrio alkaliphilus	
SB1-11Oct_0625E	SB	11	JN645436	90	Desulfobulbus propionicus	
SB1-11Oct_0625F	SB	4	GU193539	90.2	Desulfobulbus propionicus	
SB1-11Oct_0625G	SB	4	GU193577	90.1	Desulfurivibrio alkaliphilus	
SB1-11Oct_0625H	SB	4	GU193577	89.7	Desulfurivibrio alkaliphilus	
SB1-11Oct_0703A	SB	4	GU193539	89.7	Desulfurivibrio alkaliphilus	
SB1-11Oct_0703C	SB	4	GU193546	89.6	Desulfurivibrio alkaliphilus	

SB1-11Oct_0703D	SB	4	GU193560	89.6	Desulfurivibrio alkaliphilus	
SB1-11Oct_0703E	SB	4	GU193539	89.9	Desulfurivibrio alkaliphilus	
SB11Oct_0cmniH-0-19	SB	46	GU193806	91	intertidal microbial mat	Severin & Stal 2010
SB11Oct_0cmnifH-1-46_A12	SB	40	GU193810	93	intertidal microbial mat	Severin & Stal 2010
SB11Oct_0cmnifH-1-19_E06	SB	78	FJ686500	99.2	Jiaozhou Bay, China	Dang et al. 2013
SB11Oct_0cmnifH-2-46_B12	SB	44	KF847070	0.852	soil	Berthrong et al 2014
SB11Oct_0cmnifH-2-19_F06	SB	46	GU193806	82.7	intertidal microbial mat	Severin & Stal 2010
SB11Oct_0cmnifH-3-46_C12	SB	47	GU193092	88.6	intertidal microbial mat	Severin & Stal 2010
SB11Oct_0cmnifH-3-19_G06	SB	48	HM601536	83.6	Florida Bay	Olson and Lesser, unpublished
SB11Oct_0cmnifH-4-46_D12	SB	47	GU193092	94.5	intertidal microbial mat	Severin & Stal 2010
SB11Oct_0cmnifH-4-19_H06	SB	48	HM601541	83.6	Florida Bay	Olson and Lesser, unpublished
SB11Oct_0cmnifH-44-46_B01	SB	53	FR695864	89	Enrich. culture Desulfobacterium	Bergmann et al., 2011
SB11Oct_0cmnifH-45-46_C01	SB	76	JN638626	88.7	Black Sea	Kirkpatrick et al, unpublished
SB11Oct_0cmnifH-5-46_E12	SB	50	GQ441615	88.4	intertidal microbial mat	Severin & Stal 2010
SB11Oct_0cmnifH-6-46_F12	SB	77	KF846880	85.1	soil	Berthrong et al. 2014
SB11Oct_0cmnifH-7-46_G12	SB	50	GU193092	94.4	intertidal microbial mat	Severin & Stal 2010
SB24AugnifH-1-461_A12-1	SB	75	DQ176992	90.1	Narragansett Bay	Brown & Jenkins 2014
SB24AugnifH-1-474_A12-2	SB	12	KF285383	89	Desulfovibrio magneticus	
SB24AugnifH-10-469_B12	SB	51	HQ455892	93.1	South China Sea	Kong et al., 2011
SB24AugnifH-11-469_C12	SB	68	KF813035	93.9	Chesapeake Bay	Zhang et al., unpublished
SB24AugnifH-12-469_D12	SB	33	KF854518	95.2	soil	Berthrong et al. 2014
SB24AugnifH-13-469_E12	SB	33	KF854518	95.4	soil	Berthrong et al. 2014
SB24AugnifH-14-469_F12	SB	67	EF568535	89.7	Mediterranean Sea	Yogev et al. 2011 et al.
SB24AugnifH-15-469_G12	SB	33	KF854518	0.903	soil	Berthrong et al. 2014
SB24AugnifH-2-469_B11	SB	74	EF568533	89.2	Mediterranean Sea	Yogev et al. 2011 et al.
SB24AugnifH-2-461_B12-2	SB	73	HQ660939	94.1	hypoxic So. Cal Bight	Kirkpatrick et al, unpublished
SB24AugnifH-2-474_B12-1	SB	12	KF846589	79.6	soil	Berthrong et al. 2014
SB24AugnifH-3-469_C11	SB	31	JN638625	87.9	Black Sea	Kirkpatrick et al, unpublished
SB24AugnifH-3-461_C12-1	SB	68	DQ176992	90.1	Mangrove roots	Flores-Mirales et al. 2007
SB24AugnifH-3-474_C12-2	SB	29	FR695864	87.7	Desulfatibacillum alkenivorans	
SB24AugnifH-31-461_E04	SB	40	FJ756631	87.8	mid-Atlantic Coastal Ocean	Mulholland et al., unpublished
SB24AugnifH-32-461_F04	SB	40	GU193810	86.4	intertidal microbial mat	Severin & Stal 2010
SB24AugnifH-33-461_G04	SB	44	DQ177005	84.7	Mangrove roots	Flores-Mirales et al. 2007
SB24AugnifH-35-474_B09	SB	12	KF846920	89.3	soil	Berthrong et al. 2014
SB24AugnifH-39-474_F09	SB	29	HQ130019	80.8	Canadian Arctic Bay	Blais et al. 2012
SB24AugnifH-4-469_D11	SB	51	HQ455892	86.2	South China Sea	Kong et al., 2011
SB24AugnifH-4-461_D12-1	SB	72	KF854577	0.854	soil	Berthrong et al. 2014
SB24AugnifH-4-474_D12-2	SB	12	DQ176992	80.3	Mangrove roots	Flores-Mirales et al. 2007
SB24AugnifH-40-474_G09	SB	29	JX866241	88.9	Desulfovibrio magneticus	
SB24AugnifH-41-474_H09	SB	12	KF847252	88.6	Desulfovibrio magneticus	
SB24AugnifH-42-474_A10	SB	12	KF847252	89.2	Desulfovibrio magneticus	
SB24AugnifH-43-474_B10	SB	12	KF847252	89.5	soil	Berthrong et al. 2014
SB24AugnifH-5-469_E11	SB	56	KF847145	0.894	soil	Berthrong et al. 2014
SB24AugnifH-5-461_E12-1	SB	71	JN638623	86.2	Black Sea	Kirkpatrick et al, unpublished
SB24AugnifH-5-474_E12-2	SB	12	JN638704	79.2	Black Sea	Kirkpatrick et al, unpublished
SB24AugnifH-6-461_F12	SB	70	JN638625	93.7	Black Sea	Kirkpatrick et al, unpublished
SB24AugnifH-6-469_F11	SB	31	HQ660939	96.4	hypoxic So. Cal Bight	Hamersley et al. 2011
SB24AugnifH-7-469_G11	SB	69	EF568564	86.1	Mediterranean Sea	Mulholland et al., unpublished
SB24AugnifH-7-461_G12	SB	31	JN638624	93.4	Black Sea	Kirkpatrick et al, unpublished
SB24AugnifH-8-469_H11	SB	56	DQ078033	90.1	oil-contaminated marine sediment	Musat et al., 2006
SB4-11Oct_0607	SB	38	HF565512	86.9	Desulfomonile tiedjei	
SB5-11Oct_0607A	SB	57	KF285321	99.1	Narragansett Bay	Brown & Jenkins 2014
SB5-11Oct_0607B	SB	38	HQ456077	86.8	Desulfomonile tiedjei	
SB5-11Oct_0607C	SB	38	EU913058	77.1	Amazon forest	Mantilla-Paredes et al., 2009
SB5-24Aug_0625A	SB	11	DQ098249	85.8	Chesapeake Bay	Zhang et al., unpublished
SB5-24Aug_0625B	SB	11	FJ756590	86.9	mid-Atlantic Coastal Ocean	Mulholland et al., unpublished
SB5-24Aug_0625C	SB	11	JN645381	92.1	Narragansett Bay	Fulweiler et al. 2013
SB5-24Aug_0625D	SB	11	FJ756644	90.5	mid-Atlantic Coastal Ocean	Mulholland et al., unpublished
SB5-24Aug_0625E	SB	4	KF847067	94	soil	Berthrong et al. 2014
SB5-24Aug_0625F	SB	11	KF847067	0.889	soil	Berthrong et al. 2014
SB5-24Aug_0625G	SB	4	KF847067	93.9	soil	Berthrong et al. 2014
SB5-24Aug_0625H	SB	9	HM047125	99.2	Bradyrhizobium denitrificans	
SB5-24Aug_0625I	SB	9	HM047125	99.3	Bradyrhizobium denitrificans	
SB5-24Aug_0625J	SB	11	KF847067	93.7	soil	Berthrong et al. 2014
SB5-24Aug_0625K	SB	11	KF847067	89.9	Desulfocapsa sulfexigens	
SB5-24Aug_0625L	SB	9	HM047125	99.4	Bradyrhizobium denitrificans	
SB6-11Oct_0412A	SB	39	DQ098189	91.3	Desulfocapsa sulfexigens	
SB6-11Oct_0412B	SB	9	HM047125	98.9	Bradyrhizobium denitrificans	
SB6-11Oct_0412C	SB	9	HM047125	98.7	Bradyrhizobium denitrificans	
SB6-11Oct_0703A	SB	9	HM047125	99.2	Bradyrhizobium denitrificans	
SB6-11Oct_0703B	SB	9	HM047125	99	Bradyrhizobium denitrificans	
SB6-11Oct_0703C	SB	39	DQ098212	91.5	CP003985 Desulfocapsa sulfexigens	
SB6-11Oct_0704D	SB	9	HM047125	98.9	Bradyrhizobium denitrificans	
SB7JulnifH-1-52-53_A07	SB	3	JN122948	92.2	Soil Mexico	Lopez et al., unpublished
SB7JulnifH-1-52-53_A08	SB	32	GU193154	94.4	intertidal microbial mat	Severin & Stal 2010
SB7JulnifH-10-52-53_B08	SB	10	JN122948	92.4	Soil Mexico	Lopez et al., unpublished
SB7JulnifH-11-52-53_C08	SB	3	AF389780	89	saltmarsh	Lovell et al., 2001
SB7JulnifH-11-52-53_C08-2	SB	3	KF846704	90.6	soil	Berthrong et al. 2014
SB7JulnifH-11-52-53_C09	SB	45	DQ177020	92.4	Mangrove roots	Flores-Mirales et al. 2007

SB7JulnifH-12-52-53_D08	SB	3	KF846704	81.7	soil	Berthrong et al. 2014
SB7JulnifH-12-52-53_D09	SB	8	KF846671	91.9	soil	Berthrong et al. 2014
SB7JulnifH-13-52-53_E08	SB	45	GU193072	90.8	intertidal microbial mat	Severin & Stal 2010
SB7JulnifH-13-52-53_E09	SB	24	GU193093	95.4	intertidal microbial mat	Severin & Stal 2010
SB7JulnifH-14-52-53_F08	SB	3	AF389780	84.7	saltmarsh	Lovell et al., 2001
SB7JulnifH-14-52-53_F09	SB	26	DQ177038	90.1	Mangrove roots	Flores-Mirales et al. 2007
SB7JulnifH-15-52-53_G08	SB	3	GU192755	94.9	intertidal microbial mat	Severin & Stal 2010
SB7JulnifH-15-52-53_G08-2	SB	8	JN122948	92.4	Soil Mexico	Lopez et al., unpublished
SB7JulnifH-15-52-53_G09	SB	3	JN122948	91.9	Soil Mexico	Lopez et al., unpublished
SB7JulnifH-16-52-53_H08	SB	10	KF846630	81.7	soil	Berthrong et al. 2014
SB7JulnifH-16-52-53_H09	SB	28	KF846904	94.2	soil	Berthrong et al. 2014
SB7JulnifH-17-52-53_A09	SB	18	KF846865	94.7	soil	Berthrong et al. 2014
SB7JulnifH-17-52-53_A10	SB	10	JN122948	92.5	Soil Mexico	Lopez et al., unpublished
SB7JulnifH-18-52-53_B09	SB	8	GU193093	91.4	intertidal microbial mat	Severin & Stal 2010
SB7JulnifH-18-52-53_B10	SB	49	KF854519	92.5	soil	Berthrong et al. 2014
SB7JulnifH-19-52-53_C09	SB	28	KF847126	96.9	soil	Berthrong et al. 2014
SB7JulnifH-19-52-53_C09-2	SB	18	AY224028	96.3	Chesapeake Bay	Jenkins et al., 2004
SB7JulnifH-19-52-53_C10	SB	3	JN122948	92.4	Soil Mexico	Lopez et al., unpublished
SB7JulnifH-2-52-53_B08	SB	26	DQ177001	81.2	Mangrove roots	Flores-Mirales et al. 2007
SB7JulnifH-20-52-53_D09	SB	3	JN122948	92.1	Soil Mexico	Lopez et al., unpublished
SB7JulnifH-20-52-53_D09-2	SB	24	GU193792	91.6	intertidal microbial mat	Severin & Stal 2010
SB7JulnifH-20-52-53_D10	SB	26	GQ289582	90	Bradyrhizobium japonicum	
SB7JulnifH-21-52-53_E09	SB	3	JF897546	89.4	intertidal microbial mat	Severin & Stal 2010
SB7JulnifH-21-52-53_E09-2	SB	32	JN122948	84.8	Soil Mexico	Lopez et al., unpublished
SB7JulnifH-22-52-53_F09	SB	10	JN122948	92.1	Soil Mexico	Lopez et al., unpublished
SB7JulnifH-22-52-53_F09-2	SB	10	FJ395088	83.2	Salt Marsh	Gamble et al., 2010
SB7JulnifH-23-52-53_G09	SB	26	GU192755	95.6	intertidal microbial mat	Severin & Stal 2010
SB7JulnifH-23-52-53_G09-2	SB	8	DQ177038	90.1	Mangrove roots	Flores-Mirales et al. 2007
SB7JulnifH-24-52-53_H09	SB	28	KF846865	96.7	soil	Berthrong et al. 2014
SB7JulnifH-24-52-53_H09-2	SB	28	KF846625	94.4	soil	Berthrong et al. 2014
SB7JulnifH-25-52-53_A10	SB	10	FJ686502	81.7	Jiaozhou Bay, China	Dang et al. 2013
SB7JulnifH-26-52-53_B10	SB	8	GU192755	91.2	intertidal microbial mat	Severin & Stal 2010
SB7JulnifH-27-52-53_C10	SB	32	DQ177020	95.9	Mangrove roots	Flores-Mirales et al. 2007
SB7JulnifH-28-52-53_D10	SB	18	KF847126	97.2	soil	Berthrong et al. 2014
SB7JulnifH-29-52-53_E10	SB	43	DQ177038	90.6	Mangrove roots	Flores-Mirales et al. 2007
SB7JulnifH-3-52-53_C08	SB	3	JN122948	92.4	Soil Mexico	Lopez et al., unpublished
SB7JulnifH-4-52-53_D08	SB	10	JN122948	92.5	Soil Mexico	Lopez et al., unpublished
SB7JulnifH-5-52-53_E07	SB	8	KF846671	95.7	soil	Berthrong et al. 2014
SB7JulnifH-5-52-53_E08	SB	3	JN122948	92.4	Soil Mexico	Lopez et al., unpublished
SB7JulnifH-6-52-53_F07	SB	10	JN122948	92.5	Soil Mexico	Lopez et al., unpublished
SB7JulnifH-6-52-53_F07-2	SB	3	KF847181	0.825	soil	Berthrong et al. 2014
SB7JulnifH-6-52-53_F08	SB	8	GU193093	95.9	intertidal microbial mat	Severin & Stal 2010
SB7JulnifH-7-52-53_G07	SB	18	GU192755	95.4	intertidal microbial mat	Severin & Stal 2010
SB7JulnifH-7-52-53_G07-2	SB	24	KF846865	97.1	soil	Berthrong et al. 2014
SB7JulnifH-7-52-53_G08	SB	10	HM063802	81.6	Florida Bay	Olson and Lesser, unpublished
SB7JulnifH-8-52-53_H07	SB	43	GU193093	95.6	intertidal microbial mat	Severin & Stal 2010
SB7JulnifH-8-52-53_H07-2	SB	8	DQ177005	90.6	Mangrove roots	Flores-Mirales et al. 2007
SB7JulnifH-8-52-53_H08	SB	24	GU192755	95.6	intertidal microbial mat	Severin & Stal 2010
SB7JulnifH-9-52-53_A08	SB	8	GU192755	95.9	intertidal microbial mat	Severin & Stal 2010
SB7JulnifH-9-52-53_A08-2	SB	49	KF854519	0.855	soil	Berthrong et al. 2014
SB7JulnifH-9-52-53_A09	SB	26	DQ177004	81.1	Mangrove roots	Flores-Mirales et al. 2007
SB9-7Jul_0412A	SB	18	KF847132	0.947	soil	Berthrong et al. 2014
SB9-7Jul_0412B	SB	18	KF847126	0.944	soil	Berthrong et al. 2014
SLP19-24Aug_0614A	SLP	19	HM750325	92.7	Salt Marsh	Lovell & Davis 2012
SLP19-24Aug_0614B	SLP	19	HM750325	93	Salt Marsh	Lovell & Davis 2012
SLP19-24Aug_0614C	SLP	19	HM750325	93	Salt Marsh	Lovell & Davis 2012
SLP19-24Aug_0614D	SLP	19	HM750325	91.7	Salt Marsh	Lovell & Davis 2012
SLP19-24Aug_0614E	SLP	19	HM750325	92.7	Salt Marsh	Lovell & Davis 2012
SLP19-24Aug_0614F	SLP	19	HM750325	92.8	Salt Marsh	Lovell & Davis 2012
SLP19-24Aug_0614G	SLP	19	HM750325	92.8	Salt Marsh	Lovell & Davis 2012
SLP24AugnifH-1-364_A02	SLP	16	DQ098254	92.5	Chesapeake Bay	Zhang et al., unpublished
SLP24AugnifH-10-364_B03	SLP	13	KF854519	92.5	soil	Berthrong et al. 2014
SLP24AugnifH-11-364_C03	SLP	16	DQ098254	92.8	Chesapeake Bay	Zhang et al., unpublished
SLP24AugnifH-12-364_D03	SLP	13	DQ098254	92.5	Chesapeake Bay	Zhang et al., unpublished
SLP24AugnifH-15-364_G03	SLP	13	DQ098254	92.4	Chesapeake Bay	Zhang et al., unpublished
SLP24AugnifH-19-364_G01	SLP	16	KF854519	92.8	soil	Berthrong et al. 2014
SLP24AugnifH-21-368_A07	SLP	14	KF847167	90.7	Desulfovibrio vulgaris	
SLP24AugnifH-22-364_A11	SLP	16	HM750367	85.3	Salt Marsh	Lovell & Davis 2012
SLP24AugnifH-23-368_C07	SLP	30	HM601494	90.9	Desulfovibrio vulgaris	
SLP24AugnifH-24-364_C11	SLP	16	KF847073	93	Chesapeake Bay	Zhang et al., unpublished
SLP24AugnifH-24-368_D07	SLP	30	DQ098254	90.9	Desulfovibrio vulgaris	
SLP24AugnifH-25-364_D11	SLP	13	DQ098254	85.1	Chesapeake Bay	Zhang et al., unpublished
SLP24AugnifH-26-364_E11	SLP	13	DQ098254	92.7	Chesapeake Bay	Zhang et al., unpublished

SLP24AugnifH-26-368_F07	SLP	54	FJ686519	84.6	Desulfobacter curvatus	
SLP24AugnifH-27-368_G07	SLP	14	KF847073	90.6	Desulfovibrio vulgaris	
SLP24AugnifH-28-364_G11	SLP	64	CP007201	92.5	Sulfurospirillum multivorans	
SLP24AugnifH-28-368_H07	SLP	14	KF847073	90.9	Desulfovibrio vulgaris	
SLP24AugnifH-29-368_A08	SLP	16	KF847073	91	Desulfovibrio vulgaris	
SLP24AugnifH-29-364_H11	SLP	14	GU193805	86.1	intertidal microbial mat	Severin & Stal 2010
SLP24AugnifH-3-364_C02	SLP	13	DQ098254	93	Chesapeake Bay	Zhang et al., unpublished
SLP24AugnifH-30-52&53_A12	SLP	16	DQ098254	92.7	Chesapeake Bay	Zhang et al., unpublished
SLP24AugnifH-31-368_C08	SLP	14	DQ098243	90.7	Desulfovibrio vulgaris	
SLP24AugnifH-32-368_D08	SLP	14	KF846986	90.6	Desulfovibrio vulgaris	
SLP24AugnifH-33-368_E08	SLP	14	KF847167	91	Desulfovibrio vulgaris	
SLP24AugnifH-34-368_F08	SLP	30	JN122948	90.6	Desulfovibrio vulgaris	
SLP24AugnifH-35-368_G08	SLP	30	KF847167	90.9	Desulfovibrio vulgaris	
SLP24AugnifH-36-368_H08	SLP	54	FJ686519	89.1	Jiaozhou Bay, China	Dang et al., 2013
SLP24AugnifH-37-368_E06	SLP	14	KF847073	91	Desulfovibrio vulgaris	
SLP24AugnifH-39-368_G06	SLP	52	DQ177038	89.2	Mangrove roots	Flores-Mirales et al. 2007
SLP24AugnifH-4-364_D02	SLP	13	DQ098254	92.3	Chesapeake Bay	Zhang et al., unpublished
SLP24AugnifH-40-368_H06	SLP	52	DQ177038	78.5	Mangrove roots	Flores-Mirales et al. 2007
SLP24AugnifH-42-367_B04	SLP	63	HM063804	87.6	South China Sea	Kong et al., 2011
SLP24AugnifH-44-367_D04	SLP	44	CP001087	90.5	cave	Desai et al., 2013
SLP24AugnifH-45-367_E04	SLP	35	HF565511	84.9	Desulfobacter latus	
SLP24AugnifH-46-367_F04	SLP	36	HM063804	91.1	cave	Desai et al., 2013
SLP24AugnifH-47-367_G04	SLP	34	HF565512	80.8	cave	Desai et al., 2013
SLP24AugnifH-53-367_E05	SLP	35	HF565511	83.3	cave	Desai et al., 2013
SLP24AugnifH-55-367_G05	SLP	35	HF565511	91.1	cave	Desai et al., 2013
SLP24AugnifH-57-367_A06	SLP	61	FN813564	99.7	Pseudomonas stutzeri	
SLP24AugnifH-58-367_B06	SLP	60	HM063804	93.9	South China Sea	Kong et al., 2011
SLP24AugnifH-6-364_F02	SLP	27	CP001999	86.1	Arcobacter nitrofigilis	
SLP24AugnifH-7-364_G02	SLP	13	DQ098254	92.7	Chesapeake Bay	Zhang et al., unpublished
SLP29JulnifH-1-217_A10	SLP	1	DQ098249	93.6	Chesapeake Bay	Zhang et al., unpublished
SLP29JulnifH-10-217_B11	SLP	1	GU193076	93.5	intertidal microbial mat	Severin & Stal 2010
SLP29JulnifH-11-217_C11	SLP	2	DQ098249	93.6	Chesapeake Bay	Zhang et al., unpublished
SLP29JulnifH-12-217_D11	SLP	2	GU193076	86.9	intertidal microbial mat	Severin & Stal 2010
SLP29JulnifH-13-217_E11	SLP	2	GU193579	93.2	intertidal microbial mat	Severin & Stal 2010
SLP29JulnifH-14-217_F11	SLP	1	JN645429	93.5	Narragansett Bay	Fulweiler et al. 2013
SLP29JulnifH-16-217_H11	SLP	2	JN645429	91.4	Narragansett Bay	Fulweiler et al. 2013
SLP29JulnifH-17-217_A12	SLP	1	KF846820	93	soil	Berthrong et al. 2014
SLP29JulnifH-19-217_C12	SLP	1	DQ098249	93.6	Chesapeake Bay	Zhang et al., unpublished
SLP29JulnifH-2-217_B10	SLP	1	KF846820	87.5	soil	Berthrong et al. 2014
SLP29JulnifH-2-217_C01	SLP	2	GU193076	93.4	intertidal microbial mat	Severin & Stal 2010
SLP29JulnifH-20-217_D12	SLP	1	GU193836	87.2	intertidal microbial mat	Severin & Stal 2010
SLP29JulnifH-21-217_E12	SLP	1	GU193579	86.9	intertidal microbial mat	Severin & Stal 2010
SLP29JulnifH-22-217_F12	SLP	2	DQ098249	93.8	Chesapeake Bay	Zhang et al., unpublished
SLP29JulnifH-23-217_G12	SLP	2	GU193579	93.5	intertidal microbial mat	Severin & Stal 2010
SLP29JulnifH-25-217_F09	SLP	1	GU193842	87.7	intertidal microbial mat	Severin & Stal 2010
SLP29JulnifH-3-217_C10	SLP	2	GU193579	93.3	intertidal microbial mat	Severin & Stal 2010
SLP29JulnifH-3-217_D01	SLP	2	GU193842	93.5	intertidal microbial mat	Severin & Stal 2010
SLP29JulnifH-4-217_D10	SLP	2	JN645381	90.9	Narragansett Bay	Fulweiler et al. 2013
SLP29JulnifH-4-217_E01	SLP	1	GU193152	87.5	intertidal microbial mat	Severin & Stal 2010
SLP29JulnifH-5-217_E10	SLP	1	JN645381	91.4	Narragansett Bay	Fulweiler et al. 2013
SLP29JulnifH-6-217_F10	SLP	1	JN645380	91.1	Narragansett Bay	Fulweiler et al. 2013
SLP29JulnifH-6-217_G01	SLP	2	GU193152	93.5	intertidal microbial mat	Severin & Stal 2010
SLP29JulnifH-7-217_G10	SLP	1	JN645381	93.3	Narragansett Bay	Fulweiler et al. 2013
SLP29JulnifH-8-217_H10	SLP	1	GU193076	93.8	intertidal microbial mat	Severin & Stal 2010
SLP29JulnifH-9-217_A11	SLP	1	DQ098249	93.6	Chesapeake Bay	Zhang et al., unpublished
SLP8-24Aug_0412A	SLP	66	JX268325	82.6	Rice paddy	Bei et al., 2103
SLP8-24Aug_0412B	SLP	9	CP000494	99.2	Bradyrhizobium sp.	
SLP8-24Aug_0625A	SLP	6	GU193072	95.3	intertidal microbial mat	Severin & Stal 2010
SLP8-24Aug_0625B	SLP	5	FJ686527	91.9	Desulfovibrio vulgaris	
SLP8-24Aug_0625C	SLP	5	FJ686527	92.1	Desulfovibrio vulgaris	
SLP8-24Aug_0703A	SLP	5	FJ686527	92.1	Desulfovibrio vulgaris	
SLP8-24Aug_0703B	SLP	5	FJ686527	92.1	Desulfovibrio vulgaris	
SLP8-24Aug_0703D	SLP	5	FJ686527	91.8	Desulfovibrio vulgaris	
SLP8-24Aug_0703G	SLP	5	KF861215	92.2	Desulfovibrio vulgaris	
SLP8-24Aug_0703H	SLP	5	JX866366	92.1	Desulfovibrio vulgaris	
SLP8-24Aug_0703J	SLP	5	AE017286	85.3	Desulfovibrio vulgaris	
SLP8-24Aug_0703L	SLP	5	HM750649	91.9	Desulfovibrio vulgaris	
SLP8-24Aug_0703M	SLP	5	FJ686498	91.9	Desulfovibrio vulgaris	
SLP8-29Jul_0412C	SLP	55	KC243670	99.2	Calothrix sp.	
SLP8-29Jul_0607	SLP	55	KC243670	98.9	Calothrix sp.	
SLP8-29Jul_0625A	SLP	6	GU192793	95.4	intertidal microbial mat	Severin & Stal 2010
SLP8-29Jul_0625B	SLP	6	GU193045	90.3	intertidal microbial mat	Severin & Stal 2010
SLP8-29Jul_0625C	SLP	6	GU193045	95.6	intertidal microbial mat	Severin & Stal 2010
SLP8-29Jul_0625D	SLP	6	CP007031	90.5	Marichromatium purpuratum	
SLP8-29Jul_0625E	SLP	6	GU193045	95.4	intertidal microbial mat	Severin & Stal 2010
SLP8-29Jul_0625F	SLP	6	CP007031	95.1	Marichromatium purpuratum	
SLP8-29Jul_0625G	SLP	6	DQ078016	90.6	oil-contaminated marine sediment	
SLP8-29Jul_0625H	SLP	6	GU192793	90.9	intertidal microbial mat	Severin & Stal 2010
SLP8-29Jul_0625I	SLP	6	KC256769	90.3	Pseudanabaena cf.	
